# Prediction of life satisfaction from resting‐state functional connectome

**DOI:** 10.1002/brb3.2331

**Published:** 2021-08-22

**Authors:** Takashi Itahashi, Neda Kosibaty, Ryu‐Ichiro Hashimoto, Yuta Y. Aoki

**Affiliations:** ^1^ Medical Institute of Developmental Disabilities Research Showa University Tokyo Japan; ^2^ Graduate School of Advanced Mathematical Sciences Meiji University Tokyo Japan; ^3^ Department of Language Sciences, Graduate School of Humanities Tokyo Metropolitan University Tokyo Japan

**Keywords:** Human Connectome Project, intrinsic functional connectivity, life satisfaction, predictive model

## Abstract

**Background:**

Better life satisfaction (LS) is associated with better psychological and psychiatric outcomes. To the best of our knowledge, no studies have examined prediction models for LS.

**Methods:**

Using resting‐state functional magnetic resonance imaging (R‐fMRI) data from the Human Connectome Project (HCP) Young Adult S1200 dataset, we examined whether LS is predictable from intrinsic functional connectivity (iFC). All the HCP data were subdivided into either discovery (*n* = 100) or validation (*n* = 766) datasets. Using R‐fMRI data in the discovery dataset, we computed a matrix of iFCs between brain regions. Ridge regression, in combination with principal component analysis and 10‐fold cross‐validation, was used to predict LS. Prediction performance was evaluated by comparing actual and predicted LS scores. The generalizability of the prediction model obtained from the discovery dataset was evaluated by applying this model to the validation dataset.

**Results:**

The model was able to successfully predict LS in the discovery dataset (*r* = 0.381, *p* < .001). The model was also able to successfully predict the degree of LS (*r* = 0.137, 5000‐repetition permutation test *p* = .006) in the validation dataset, suggesting that our model is generalizable to the prediction of LS in young adults. iFCs stemming from visual, ventral attention, or limbic networks to other networks (such as the dorsal attention network and default mode network) were likely to contribute positively toward predicted LS scores. iFCs within ventral attention and limbic networks also positively contributed to predicting LS. On the other hand, iFCs stemming from the visual and cerebellar networks to other networks were likely to contribute negatively to the predicted LS scores.

**Conclusion:**

The present findings suggest that LS is predictable from the iFCs. These results are an important step toward identifying the neural basis of life satisfaction.

## Graphical abstract

Intrinsic functional connectivities (iFCs) successfully predicted life satisfaction (LS). iFCs stemming from visual, ventral attention (VAN), or limbic networks to other networks were likely to contribute positively toward predicted LS scores, while iFCs within VAN and limbic networks also positively contributed to predicting LS. On the other hand, iFCs stemming from the visual and cerebellar networks to other networks were likely to contribute negatively to the predicted LS scores.

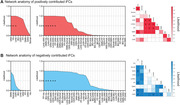

## 1 INTRODUCTION

Good subjective well‐being (SWB) is protective against the psychological impacts of major life events and is related to longevity and a lower chance of physical illness (Diener & Chan, 2011; Luhmann et al., 2012; Wood & Joseph, 2010). Because of these advantages, achieving better SWB has been an important issue for policymakers since the 1980s (Diener, 1984; Layard, 2010). However, because of its complex multifaceted nature, improving SWB is not easy. One of the most important components of SWB is life satisfaction (LS), defined as “a cognitive and global evaluation of the quality of one's life as a whole” (Pavot & Diener, 2008). LS is associated with a wide variety of psychological and psychiatric sequelae, such as self‐esteem (Diener & Marissa, 2009) and even suicide (Koivumaa‐Honkanen et al., 2001).

Despite such importance, LS itself is complex and changes with life course; thus, only a few studies have examined its neural basis (Kong, Ding, et al., 2015; Waldinger et al., 2011). A morphometric study showed that LS is positively associated with regional gray matter volume in the right parahippocampal gyrus and negatively correlated with gray matter volume in the left precuneus and ventromedial prefrontal cortex (Kong, Ding, et al., 2015). A functional magnetic resonance imaging (fMRI) study showed that higher LS is associated with stronger functional connectivity within the emotion‐processing network (Waldinger et al., 2011). These prior studies showed that unique neural correlates of LS can be found within their respective participant groups. However, a generalizable common neural basis for LS has not yet been conclusively demonstrated.

Given the importance of LS to SWB, the consequences of low LS, and the relevance of LS to individuals worldwide, whether or not LS has a generalizable neural basis should be determined. In the present study, we examine whether a predictive model for LS can be constructed from intrinsic functional connectivity (iFC) data and subsequently examine whether the neural basis of LS in one group of healthy adults is generalizable to a larger, separate healthy adult group.

## 2 MATERIALS AND METHODS

### 2.1 Human Connectome Project dataset

We used the Human Connectome Project (HCP) Young Adult dataset, comprising MRI data collected from 1200 young adult participants (22−35 years old) (Glasser et al., 2013). We then removed subjects who did not complete two resting‐state fMRI (R‐fMRI) sessions, for whom psychological well‐being measures were not obtained, or who exhibited excessive head motions during the MRI scans. After these exclusion criteria, 866 subjects remained. Because this dataset contained biological siblings, there was a possibility for biasing the prediction model due to the genetic and shared environmental factors among siblings. The HCP dataset provided a list of subjects unrelated to other subjects. To avoid undesirable information leakage in constructing the prediction models, we used 100 unrelated subjects (male: *n* = 46, female: *n* = 54, LS: 54.47 ± 9.00 [mean ± SD]) out of the 866 for the discovery dataset. The remaining 766 subjects were used for the validation dataset (male: *n* = 363, female: *n* = 403, LS: 54.79 ± 8.89 [mean ± SD]). The degree of general LS in HCP participants was measured using the National Institutes of Health (NIH) toolbox (Salsman et al., 2014). We note that the overarching aim of the current study is building the generalizable model to predict LS. Thus, we eschewed adjusting potential confounding demographic factors, such as race and income. In other words, we intended to accept heterogeneity of the participants, while aiming to achieve the acceptable prediction performance.

### 2.2 R‐fMRI data processing and network construction

We obtained minimally preprocessed R‐fMRI data from the publicly available HCP database (Glasser et al., 2013). Using an in‐house MATLAB code, we applied additional processing procedures to the R‐fMRI data, including the removal of the first 10 s of data for each run and nuisance regression on the data. Nuisance regressors comprised linear detrending, six head motion parameters, and averaged signals from subject‐specific white matter, ventricle, and gray matter masks, as well as their derivatives. A band‐pass filter (0.008−0.1 Hz) was then applied to the residuals. Frame‐wise displacement (FD) was calculated to identify motion‐contaminated volumes, and a scrubbing method with an FD threshold of 0.5 mm was applied to reduce spurious changes in iFCs due to subtle head motion during the scans (Power et al., 2012 ).

We characterized each individual's whole‐brain functional connectome using 427 regions of interest (ROIs): 400 surface‐based cortical regions (Schaefer et al., 2018), 17 subcortical regions (Fischl et al., 2002), and 10 functionally parcellated cerebellar regions (King et al., 2019). Pearson correlation coefficients were calculated between all possible pairs of ROIs, yielding a 427 × 427 iFC matrix for each participant. Fisher's *r*‐to‐*z* transform was further applied to each correlation coefficient. These procedures yielded 90,951 unique iFCs, excluding the diagonal elements of the iFC matrix. The lower triangular portion of the iFC matrix was vectorized and concatenated across participants, resulting in a 100 × 90,951 feature matrix. To improve the interpretability of our findings, we characterized the 427 ROIs into nine resting‐state network (RSN) labels comprising seven RSNs previously described (Yeo et al., 2011), as well as basal ganglia (BG) and cerebellar (CER) networks.

### 2.3 Constructing the prediction model for LS using iFCs

**FIGURE 1:**
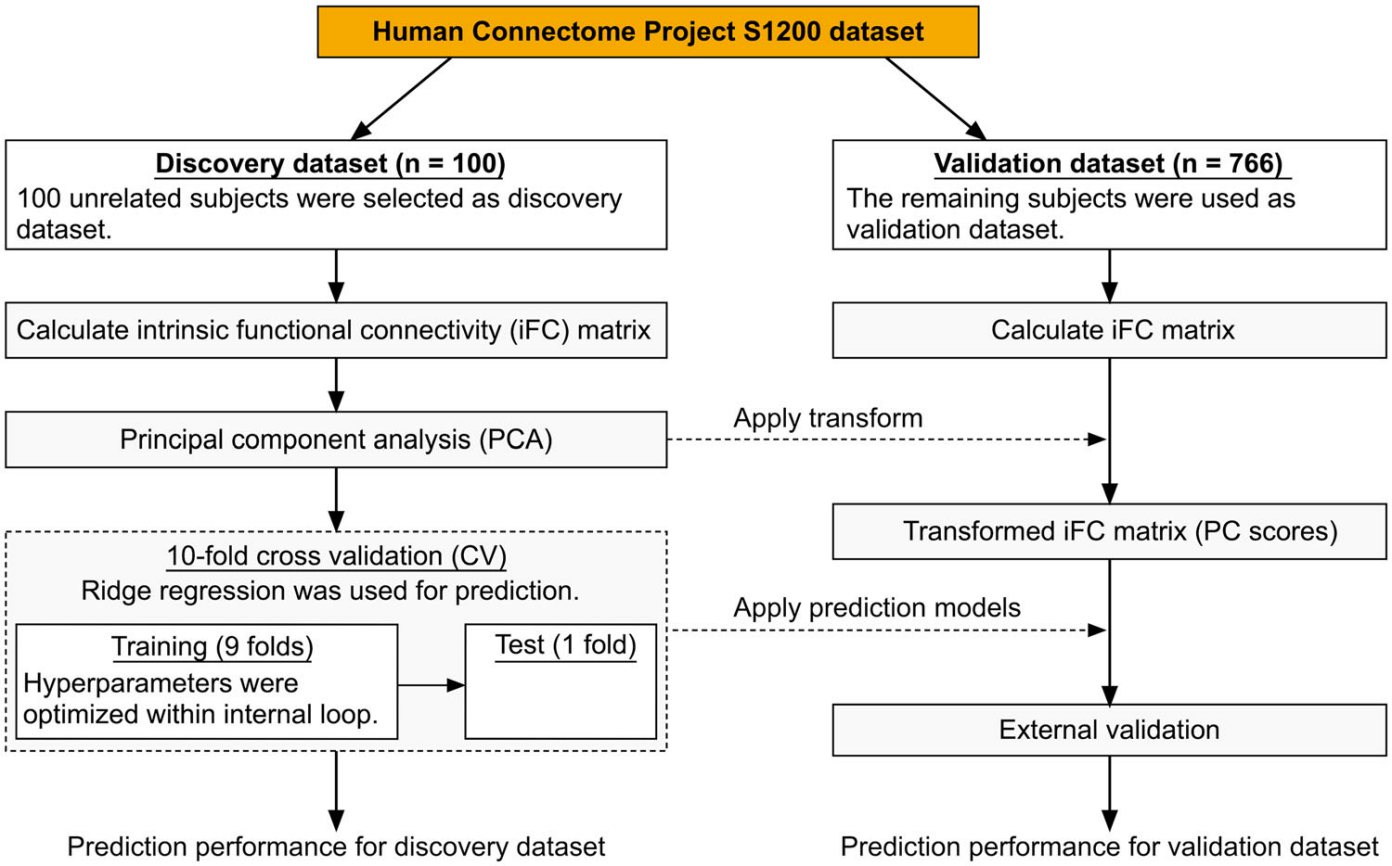
Schematic diagram of the procedure for constructing the life satisfaction (LS) prediction model and assessing its predictive power

To test the association between iFC and the degree of LS, we constructed a prediction model for LS using ridge regression in combination with principal component (PC) analysis (PCA) and a 10‐fold cross‐validation (CV) approach (Hoerl & Kennard, 1970). As shown in Figure 1, we first applied PCA to the discovery dataset to reduce the dimensionality of features into 99 PC scores. The transformation matrix obtained from the discovery dataset was also applied to the validation dataset, which, it should be noted, had not been used to obtain the transformation matrix. Then, the discovery dataset was divided into 10 groups, or folds, for 10‐fold CV. We used nine folds to construct the ridge regression prediction model, and the remaining fold was used to test the efficacy of the constructed model. The hyperparameter was optimized within the internal loop; that is, the test fold data were not used to optimize the hyperparameter. To evaluate the prediction performance of the obtained model, we calculated the Pearson correlation coefficient between averaged predicted and actual scores.

### 2.4 Permutation test for assessment of prediction performance

To assess the statistical significance of the performance of the model in predicting LS, we used a permutation test with 5000 iterations. Briefly, we shuffled LS scores at each iteration and constructed a prediction model with 10‐fold CV as described above. To assess the null models’ prediction performance, we computed the Pearson correlation coefficient between predicted and actual scores, yielding a null distribution from which the *p* value could be calculated. Statistical significance was set to *p* < .05.

### 2.5 Generalization of the prediction model for the validation dataset

The generalizability of the prediction model was tested using the validation dataset (*n* = 766). As we had obtained 10 prediction models from 10‐fold CV, we applied these prediction models to the validation dataset and computed the Pearson correlation coefficient between the averaged predicted and actual scores. Again, we constructed the null distribution using prediction models obtained by permutation test with 5000 iterations. Statistical significance was set to *p* < .05.

### 2.6 Contribution of iFCs to predicted LS

We measured the extent to which iFCs contributed to the prediction of LS by calculating the weight contribution of each iFC. As both PCA and ridge regression are linear methods, the contribution of each iFC was calculated by multiplying the PCA‐derived transformation matrix and the regression coefficient matrix of our prediction models, yielding 10 weights for each iFC. The contribution of each iFC was assessed using one‐sample *t*‐tests, with statistical significance set to *p* < .05 adjusted with a Bonferroni correction for multiple comparisons (90,951 comparisons).

To further improve the interpretability of our findings, we examined network anatomy in a similar manner to previous studies (Barron et al., 2020; Lake et al., 2019). Briefly, we computed the probability that iFCs are shared between the networks identified by our prediction model and within or between nine canonical RSNs. Statistical significance was determined using a hypergeometric cumulative distribution function after applying Bonferroni correction for 45 comparisons.

## 3 RESULTS

### 3.1 Prediction performance in the discovery dataset

**FIGURE 2:**
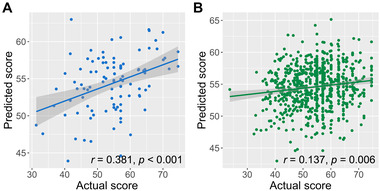
Scatter plots of actual versus model‐predicted life satisfaction (LS) scores. For the discovery (a) and validation (b) datasets, Pearson correlation coefficients were calculated between actual and predicted LS scores. Permutation tests with 5000 iterations were conducted to examine the statistical significance. Statistical threshold was set to *p* < .05

The model was able to successfully predict the degree of LS reported in healthy young adults by using PC scores derived from iFCs (*r* = 0.381, 5000‐repetition permutation test *p* < .001) in the discovery dataset of 100 individuals (Figure 2a).

### 3.2 Prediction performance in the validation dataset

Having determined that our obtained model could successfully predict the degree of LS in the discovery dataset, we applied this prediction model to the validation dataset. As we obtained 10 models through the 10‐fold CV, we applied each of these models to the validation dataset and considered their averaged scores as the predicted scores for the validation dataset. The model was also able to successfully predict the degree of LS (*r* = 0.137, 5,000‐repetition permutation test *p* = .006) in the validation dataset of 766 individuals, suggesting that our model is generalizable to the prediction of LS in young adults (Figure 2b).

### 3.3 Contribution of iFCs to the prediction performance

**FIGURE 3:**
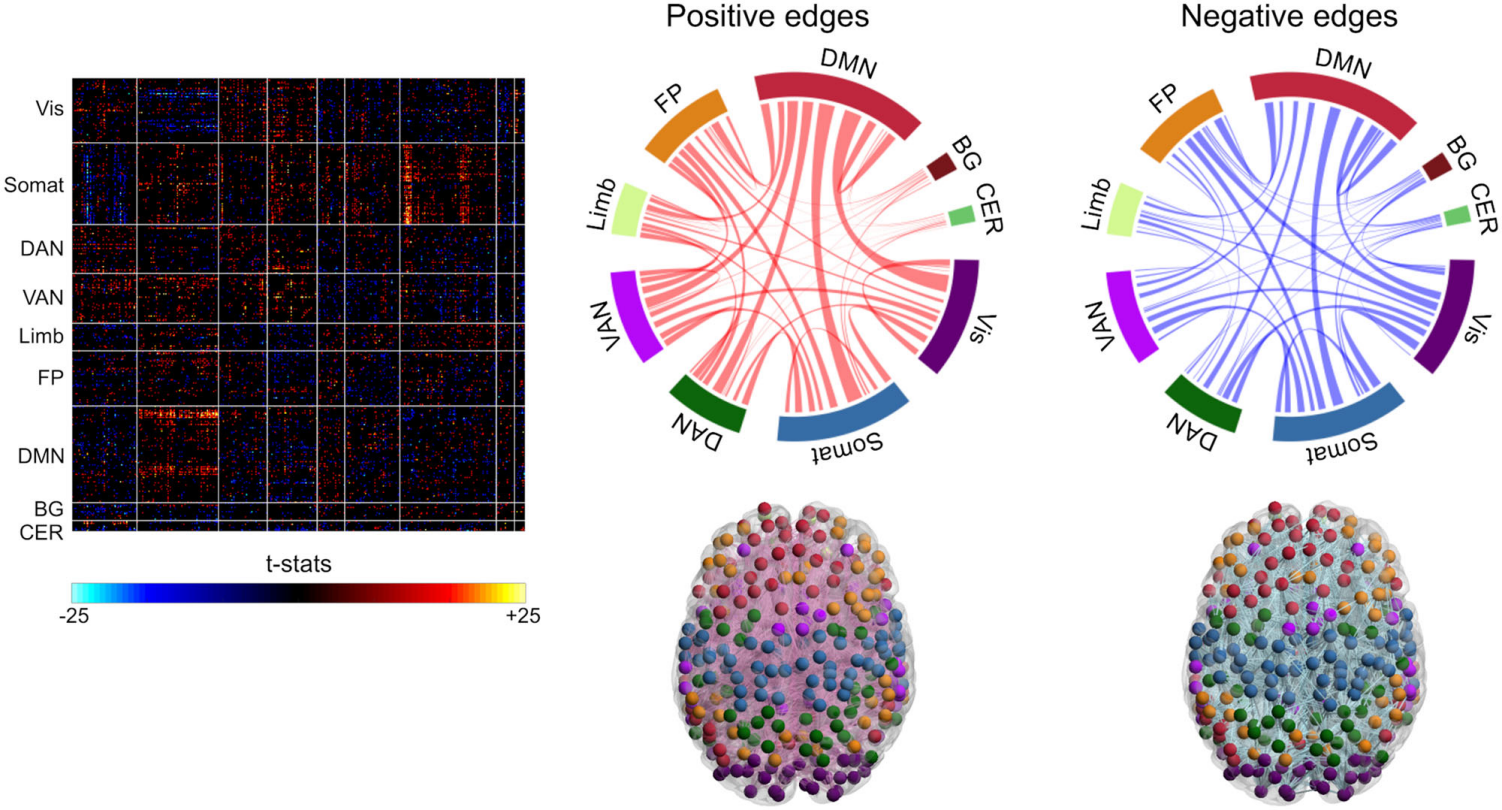
Overview of intrinsic functional connectivities (iFCs) contributing to the prediction of life satisfaction. The left panel shows the matrix, each element of which represents *t*‐statistics. The red and blue colors indicate that iFCs positively and negatively contributed to predict the life satisfaction (LS) score, respectively. The middle and left upper panels show the connectogram of iFCs contributing to the prediction of LS scores. The middle and left bottom panels show the brain maps

We computed the contribution of each iFC by multiplying the PCA‐derived transformation matrix and the weights of prediction models. Statistical analyses revealed that 6164 iFCs were significant contributors to the prediction of LS (*p* < .05/90,951), comprising 3479 iFCs contributing positively to predicted LS scores and 2685 iFCs contributing negatively to predicted LS scores (Figure 3). Qualitatively, iFCs stemming from the default mode network (DMN) were the dominant contributors to the prediction of LS.

**FIGURE 4:**
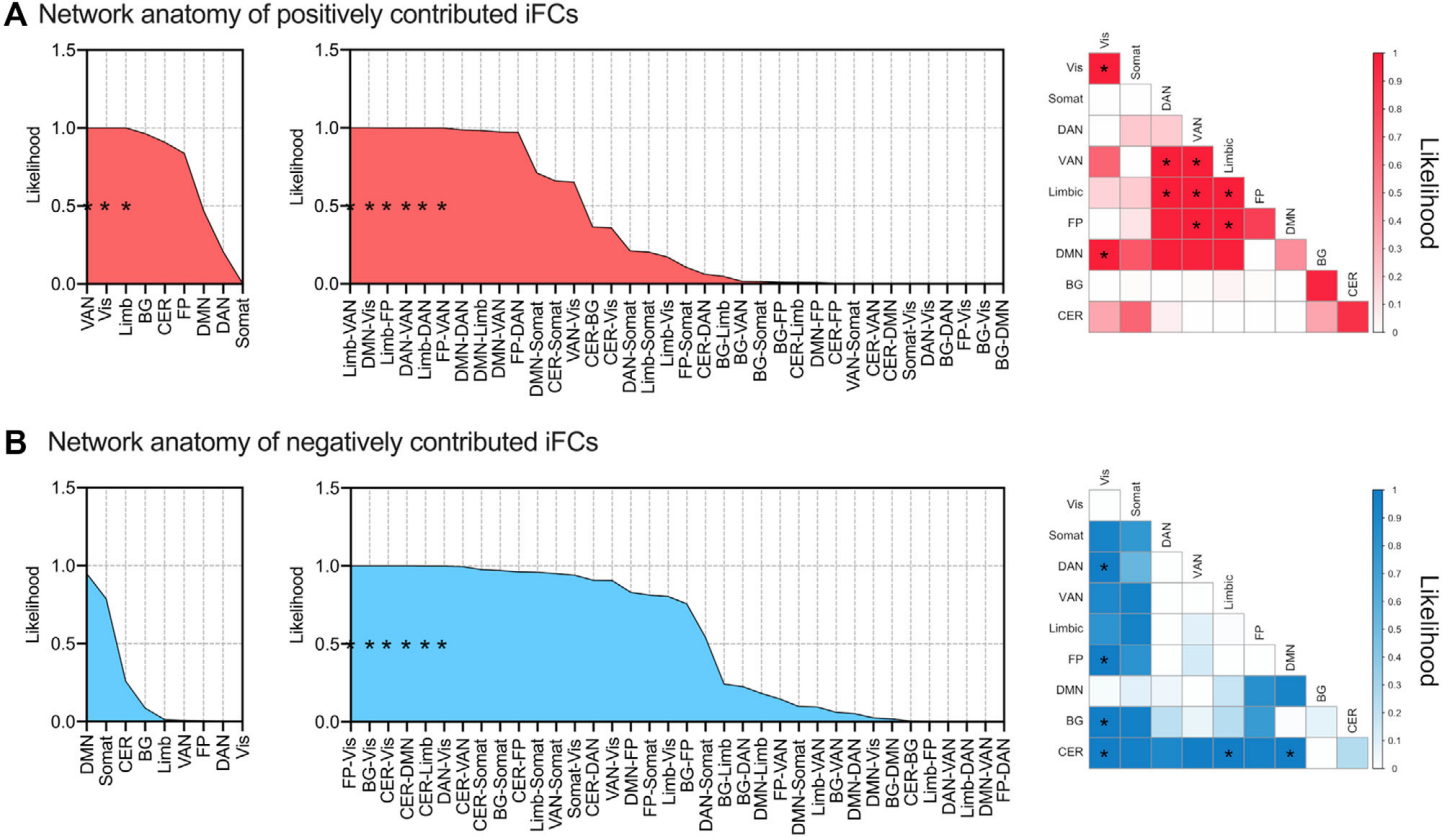
Network anatomy of prediction model. For (a) positively and (b) negatively contributing intrinsic functional connectivities (iFCs), edge overlap within and between nine canonical resting‐state networks (RSNs) was plotted. Each plot shows the likelihood (1 − *p* value) estimated from the probability of edges shared between canonical RSNs and the prediction model. In all plots, the nine within‐network (left panel) and 36 between‐network (middle panel) pairs are sorted in descending order of probability. The right panel shows the interaction of networks. The asterisk represents the statistical significance after Bonferroni correction for multiple comparisons (i.e., *p* < .025/45). Abbreviations: BG, basal ganglia; CER, cerebellar; DAN, dorsal attention network; DMN, default mode network; FP, fronto‐parietal; Limb, limbic; Somat, somatomotor; VAN, ventral attention network; Vis, visual

To further examine the model's network anatomy, we computed *p* values between our network and within or between nine canonical RSNs (with a significance level of *p* < .025/45) for iFCs contributing positively and negatively to LS prediction. Among iFCs contributing to LS, iFCs stemming from visual, ventral attention (VAN), or limbic networks to other networks (such as the dorsal attention network [DAN] and DMN) were likely to contribute positively toward predicted LS scores (Figure 4a). iFCs within VAN and limbic networks also positively contributed to predicting LS. On the other hand, iFCs stemming from the visual and cerebellar networks to other networks (such as the DAN, DMN, BG, and limbic networks) were likely to contribute negatively to the predicted LS scores (Figure 4b).

## 4 DISCUSSION

Analyzing a sizable R‐fMRI dataset in adults, we demonstrated that LS scores in healthy young adults could be predicted by a model based on iFC. Furthermore, the LS prediction model constructed using data from one group of healthy people could be used to predict the LS of another group of healthy people, suggesting a generalizable neural basis for LS. To achieve this novel finding, we first constructed a prediction model using iFCs of 100 healthy participants. We applied PCA and 10‐fold CV in the prediction model, which were then applied to the validation dataset. Although the correlation between the actual and predicted scores was weaker in the validated dataset than that in the discovery dataset (Figure 2), the findings showed high generalizability of the prediction model.

Significantly, the present study established a protocol to predict LS from R‐fMRI. This research was inspired by connectome‐based predictive modeling (CPM), which uses large‐scale neuroimaging data to predict individual differences in traits and behavior (Shen et al., 2017). Utilizing CPM, a number of studies have successfully predicted attention, anxiety, and mother–infant bonding (Ren et al., 2021; Rutherford et al., 2020; Yoo et al., 2018). In line with the conclusions of these prior studies, the present findings suggest that changes in functional connectivity may improve LS. Additionally, the present results provide insight into the functional connectivity pathways on which we should focus. Future research could investigate potential mechanisms of intervention to enhance LS.

The current findings should be interpreted in the context of several prior neuroimaging studies that examined the brain‐LS relationships. Kong, Wang, et al. (2015) showed that LS can be predictable with regional homogeneity (ReHo). Kong et al. and the current study share a part of findings. Namely, they identified that dorsal anterior cingulate cortex (ACC) was related to LS, while the dorsal ACC was included in the current list of nodes. However, there are some important differences in the assumption. First, we did not intend to identify one brain region or a few as a neural correlate for LS, but we did assume that iFC pattern would serve as a neural correlate of LS. Second, we adopted iFC as a marker for the LS because we assumed that not only short‐distance but also long‐distance connectivity would also work as neural correlates. In contrast, ReHo focuses on the local connectivity. Another study by Waldinger et al. (2011) adopted functional connectivity, but they focused on some brain regions by setting ROIs. However, they split the participants based on LS into high LS and low LS groups. In contrast, the current study assumed that LS was not categorical but dimensional. In this context, we identified a large number of iFCs contributing significantly to the prediction of LS (Figure 3), despite employing stringent corrections for multiple comparisons. The number of iFCs significantly involved in LS prediction is most extensive in the DMN, which may be driven by the fact that the DMN possesses the largest number of ROIs. Indeed, at the network level, the DMN did not frequently reach statistical significance (Figure 4). Instead, the VAN and limbic system both showed a significant contribution to LS. Given that the VAN and limbic system play roles in reorienting the attention (Vossel et al., 2014 ) and in motivation and emotional processing (Mogenson et al., 1980), these components of cognition may be associated with LS. It should be noted that these networks comprise several structures, that is, nodes in the present analyses. However, we do not delve into which nodes in these networks are involved, as this is beyond the scope of the present study; additionally, the large‐scale brain network concept assumes that the network per se serves as the neural basis for these cognitive components. The present findings are indirectly consistent with prior psychological studies that reported a link between LS and emotion and attention (Bastian et al., 2014; Diener et al., 2012). However, LS is not a simple combination of emotion and attention but is more complex and is related to culture, society, and the environment (Chen et al., 2015; Schimmack et al., 2002). In this regard, we need to emphasize that although the present study aimed to show the generalizability of our prediction model for LS, the current study did not overcome the impact of the difference in culture, society, and the environment as the data were obtained in a single‐center in the states. Future large‐scale international collaborative study is expected to overcome the limitation.

The present findings have some more limitations. First, we used a cross‐sectional dataset to examine the associations between iFCs and LS. Although we used the validation dataset to confirm the main findings, the causal relationship between iFCs and LS remains unclear. Future longitudinal investigations are necessary to elucidate causal relationships. Second, LS scores were self‐reported, which may introduce bias. However, LS is intrinsically self‐reported as a measure of how he or she subjectively feels, regardless of objective evaluation by others. Finally, although we paid much attention to avoiding undesirable information leakage in constructing the prediction model, demographic characteristics that might potentially be related to LS, such as life events (Luhmann & Eid, 2009), medical status and religiosity (Levin et al., 1995), in the present participant groups were not available. However, given that the present study aimed to show the generalizability of the prediction model, the potential heterogeneity of the present participants would further support the generalizability of our model.

## 5 CONCLUSION

We successfully predicted LS in unrelated healthy young adults using a prediction model constructed from R‐fMRI data. Additionally, the obtained model successfully predicted the LS scores in a further validation dataset, suggesting that LS has generalizable neural basis. The present findings are a step toward future intervention strategies to enhance LS, which could potentially bring the wide variety of benefits associated with good SWB to people worldwide.

## CONFLICT OF INTEREST

The authors declare no conflict of interest.

### PEER REVIEW

The peer review history for this article is available at https://publons.com/publon/10.1002/brb3.2331

## Supporting information

Supporting Information

## Data Availability

The authors declare that the data supporting the findings of this study are available within the article with DOI 10.1016/j.neuroimage.2013.04.127
