# Supplementary material for: Prediction of life satisfaction from resting‐state functional connectome
Source: Brain Behav. 2021 Aug 22;11(9):e2331. doi: 10.1002/brb3.2331 (PMC8442592; doi:10.1002/brb3.2331)
Supplement: Supplementary file 1 — Supporting Information [file BRB3-11-e2331-s001.docx]

**Supplementary Information**

**Characterization of network anatomy**

To improve the interpretability of our findings, we examined the network anatomy in a similar manner to previous studies (Barron et al., 2020; Lake et al., 2019). Once the contributions of intrinsic functional connectivity (iFCs) were identified using one-sample t-tests with statistical significance set to *P* < 0.05 adjusted with Bonferroni correction for multiple comparisons. These procedures identified 3,479 iFCs contributing positively to predicted LS scores, while 2,685 iFCs contributing negatively to predicted LS scores (see **Results** in the main text).

For each set of iFCs contributing to predicted LS scores, we computed the probability that iFCs are shared between the networks identified by our prediction model and nine canonical resting-state networks (RSNs). P-values were computed using the hypergeometric cumulative density function (hygecdf) implemented in MATLAB as follows:

$$P=1-\text{hygecdf}\left( x,M,K,n \right),$$

where *x* stands for the number of overlapping iFCs between our network and within or between canonical network. The variable, *M*, is equivalent to the number of total iFCs in the whole brain (i.e., *M* = 90,951), while the variables, *n* and *K*, equals to the total numbers of iFCs in our network of interest and the canonical network of interest, respectively. Statistical significance was set to P < 0.05 adjusted with Bonferroni correction for multiple comparisons (e.g., 45 comparisons).

**Robustness of prediction model for life satisfaction (LS)**

To confirm the robustness of our findings, we performed an additional analysis using split-half procedure. In this analysis, we merged all the subjects as one dataset, and then we randomly split subjects into two datasets, each of which contained 433 subjects. We regarded the first dataset as discovery dataset and the second one as validation dataset. Using Similar to the main analysis, we constructed the prediction model using the discovery dataset. The constructed prediction model was then applied to the validation dataset. The Pearson correlation coefficient between the actual and predicted scores were then computed. Statistical significance was evaluated using permutation tests with 1,000 iterations at each split-half procedure. These procedures were repeated 100 times.

Figure S1 shows the prediction performance using split-half procedure. Although the prediction performance was decreased when compared to our main findings, the models were still able to predict the degree of LS scores in the discovery dataset (*r* = 0.21±0.01 [mean ± standard deviation (SD)], *P* < 0.05). The models were also able to predict the degree of LS scores in the validation dataset (*r* = 0.17±0.02 [mean ± standard deviation (SD)], *P* < 0.05). These results suggest that our findings were robust to the selection of participants.


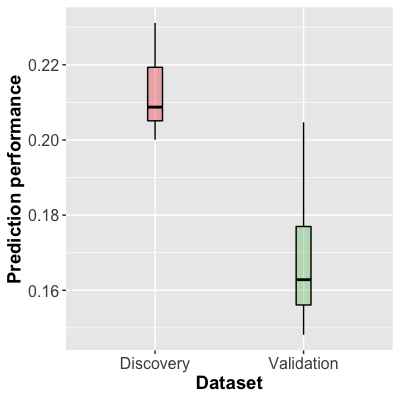


**Figure S1. The results of prediction performance using split-half procedure.**

The models were constructed using split-half procedure with 100 times. The prediction performance was evaluated using the Pearson correlation coefficient between the actual and predicted LS scores. Statistical significance was evaluated using permutation tests with 1,000 iterations in each split-half procedure.
